# Evaluation of recurrent ventricular tachyarrhythmias in patients who survived out-of-hospital cardiac arrest due to ventricular fibrillation: eligibility for subcutaneous implantable defibrillator therapy

**DOI:** 10.1007/s10840-018-0490-4

**Published:** 2018-11-26

**Authors:** Dominic A. M. J. Theuns, Rohit E. Bhagwandien, Tamas Szili-Torok, Felix Zijlstra, Sing-Chien Yap

**Affiliations:** Department of Cardiology, Erasmus MC, Room RG-632, PO Box 2040, 3000 CA Rotterdam, The Netherlands

**Keywords:** Cardiac arrest, Ventricular fibrillation, Subcutaneous ICD, Appropriate shocks, Patient selection, Antitachycardia pacing

## Abstract

**Purpose:**

The subcutaneous implantable defibrillator (S-ICD) was developed to avoid complications related to transvenous leads. A trade-off with the S-ICD is the inability to deliver antitachycardia pacing (ATP). Data is scarce about the recurrence and characteristics of ventricular tachyarrhythmias (VTa) during a follow-up in survivors of out-of-hospital cardiac arrest due to ventricular fibrillation (OHCA-VF). The aim of the study is to determine the characteristics of VTa triggering ICD therapy in order to assess whether survivors of OHCA-VF are eligible candidates for the S-ICD.

**Methods:**

All OHCA-VF patients who received a transvenous ICD were identified, 378 patients, age 57 ± 14 years, predominantly male (76%) with ischemic heart disease (58%). Arrhythmic endpoints were appropriate ICD therapies for any ventricular arrhythmia.

**Results:**

Over a median follow-up of 4.5 years, 690 VTa in 91 patients (24%) were terminated by ICD therapy; 70% of patients had < 5 VTa with ICD therapy. VTa with cycle length ≤ 300 ms were mainly (82%) treated by shock, while 83% of VTa with cycle length > 300 ms were treated by ATP. The presence of a remote myocardial infarction (OR 2.07; 95% CI 1.08–3.97) and LVEF ≤ 0.35 (OR 2.09; 95% CI 1.09–4.00) were significantly associated with the occurrence of VTa with cycle length > 300 ms.

**Conclusion:**

S-ICD implantation may be reasonable in survivors of OHCA-VF who present without a remote myocardial infarction and LVEF > 35%.

## Introduction

Placement of an implantable cardioverter-defibrillator (ICD) is a class 1A indication for survivors of cardiac arrest due to ventricular fibrillation (VF). These recommendations are based on the results of randomized clinical trials [1–4]. ICD implantation in the setting of secondary prevention has been labeled as “appropriate use” in the recent Appropriate Use Criteria Task Force report [5]. Despite the effectiveness of the ICD in terminating life-threatening ventricular arrhythmias, contemporary transvenous ICD systems have been associated with acute and chronic complications due to the use of transvenous leads [6, 7]. Based on this, an entirely subcutaneous implantable defibrillator (S-ICD) has been developed as an alternative to the transvenous ICD system [8]. The S-ICD-system holds the promise of life-saving defibrillation [9–11]. However, a trade-off with the S-ICD is the inability to deliver antitachycardia pacing (ATP). The question arises “who are candidates for the S-ICD system?”; one group is those patients who are young and are, therefore, at greater lifelong risk of complications from transvenous systems. Another opinion is that the S-ICD should be limited to patients for primary prevention of sudden death. Is secondary prevention of sudden death a contra-indication for the S-ICD? Given the inability to deliver ATP, the S-ICD is contra-indicated in secondary prevention patients with a history of monomorphic ventricular tachycardia (VT). But little is known about the recurrence and characteristics of ventricular arrhythmias during follow-up in survivors of cardiac arrest due to VF (OHCA-VF).

The aim of the study is to determine characteristics of ventricular arrhythmias triggering ICD therapy in order to assess whether survivors of OHCA-VF are eligible candidates for the S-ICD.

## Methods

### Study population

Patients for this retrospective observational cohort study were obtained from the prospectively collected registry of all patients who underwent ICD implantation at the Erasmus Medical Center, Rotterdam, The Netherlands. For the purpose of the study, patients with ischemic or nonischemic heart disease who survived OHCA-VF were included. Patients with age < 18 years or inherited arrhythmia disorders were excluded from analysis. The study period for inclusion was from January 2000 to June 2015. The administrative censoring date for analyses was December 2015 for all patients alive until that date.

Data on baseline clinical characteristics, implantation procedures, and scheduled or unscheduled follow-up visits were prospectively collected in the Erasmus MC ICD registry. Information on clinical variables was acquired at the time of ICD implantation. For all patients, renal function was assessed by estimating the baseline glomerular filtration rate (eGFR) using the abbreviated Modification of Diet in Renal Disease (MDRD) Study equation [12]. Impaired renal function was defined as an eGFR < 60 ml/min/1.73m^2^ according to practice guidelines [13]. The elapsed time from most recent myocardial infarction to ICD implantation was dichotomized at 18 months based on the results of the MADIT II study [14]. A remote myocardial infarction was defined as the time between most recent myocardial infarction and ICD implantation of ≥ 18 months. The programming of the VT zone of the ICD was set to 330–350 ms and the VF zone was set to 250–300 ms. Over the years, the programming of devices has changed, current programming strategies apply higher rate cutoffs and longer duration for arrhythmia detection. In order to identify possible trends, we defined 3 groups according to the implant year (1, 2000–2004; 2, 2005–2009; 3, 2010–2015).

### The follow-up and ICD therapy event analysis

The follow-up started at the time of ICD implantation. Device interrogation was performed on regular visits scheduled every 3- to 6-month basis, and after symptomatic events. At each visit, arrhythmic events with stored electrograms (EGMs) were retrieved from the device’s memory. Two investigators analyzed the stored EGMs with therapy delivery to classify the arrhythmia and assess the appropriateness of device classification and therapy. Ventricular tachyarrhythmias were defined as events with a sudden increase in rate combined with a change in ventricular near-field and far-field EGM morphology from the baseline rhythm. If an atrial EGM was present, the presence of atrioventricular dissociation (ventricular rate > atrial rate) was used to diagnose ventricular tachyarrhythmia. The differentiation of polymorphic from monomorphic ventricular tachyarrhythmias was based on regularity in the rhythm and in the morphology of the near-field and far-field EGMs. Polymorphic ventricular tachyarrhythmias had irregularity in the rhythm and changing morphology in near-field and far-field EGMs. Appropriate ICD therapy was defined as ATP or shock delivered for a ventricular tachyarrhythmia.

Survival status of patients was retrieved from the civil registry. As required by Dutch law, all deceased inhabitants must be registered at the civil registry within 3 days. For analysis of the endpoints, the maximum follow-up was set at 10 years. For patients whose implant date was more than 10 years before December 2015, events occurring after 10 years were not used in the analysis.

### Statistical analysis

Normality of distribution as assessed using the Shapiro-Wilk test. Continuous variables are presented as mean ± SD or as median with 25th and 75th percentiles, where appropriate. Data were compared by the ANOVA or Kruskal-Wallis test, as appropriate. Categorical data are expressed as percentages and compared with Fisher’s exact test. Cumulative mortality and event rates of appropriate ICD therapy were calculated according to the Kaplan-Meier method and were compared with the log-rank test. Univariate predictors associated with appropriate ICD therapy were included in a multivariate logistic regression model**.** Odds ratios (OR’s) are presented with corresponding 95% confidence intervals (CI’s). Statistical analysis was performed using STATA version 12 SE for Windows (StataCorp, College Station, TX) and SPSS version 24 (IBM Corp., Somers, NY). Statistical significance was defined as *P* < 0.05 (two-tailed).

## Results

### Study population

The study cohort consisted of 378 patients. The population was predominantly male (76%) with a mean age of 57 ± 14 years. The majority of patients had ischemic heart disease (58%) and the mean LVEF was 40 ± 16%, with 154 patients (41%) having a LVEF ≤ 35%. Further baseline characteristics of the study population are presented in Table 1. The median follow-up was 4.5 years (2.0 to 7.6 years), during which 91 patients (24%) received appropriate device therapy (ATP or shock) and 66 patients (18%) died.

**Table 1:** Demographics and clinical characteristics at baseline

	Total (*n* = 378)
Demographics	
Age, y	57 ± 14
Male gender	286 (76%)
Clinical characteristics	
Ischemic heart disease	218 (58%)
Myocardial infarction	171 (45%)
History of CABG	67 (18%)
History of PCI	120 (32%)
Dilated cardiomyopathy	49 (13%)
NYHA class > II	25 (7%)
Ejection fraction, %	40 ± 16
QRS duration, ms	110 (98–134)
Diabetes mellitus	50 (13%)
eGFR, ml/min/1.73m^2^	83 ± 27
eGFR < 60 ml/min/1.73m^2^	67 (18%)
Pharmacological therapy	
Amiodarone	56 (15%)
Beta blocker	284 (75%)
Digoxin	35 (9%)
ACE inhibitor	259 (69%)
Diuretic	157 (42%)
Statin	204 (54%)

Continuous data are presented as mean ± SD or median and corresponding 25th and 75th percentiles

*ACE*, angiotensin converting enzyme; *eGFR*, estimated glomerular filtration rate; *NYHA*, New York Heart Association

### The first appropriate ICD intervention

**Fig. 1:**
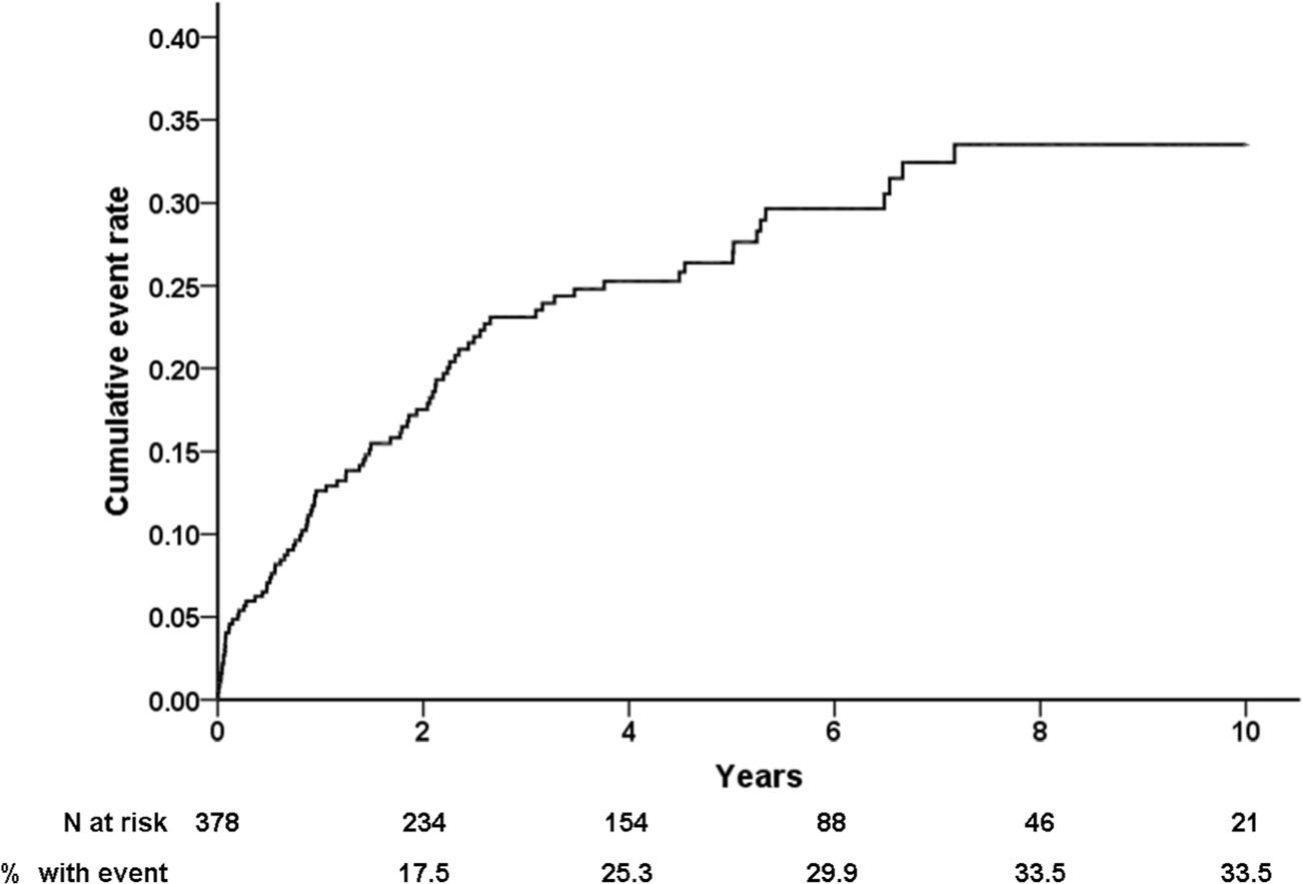
Cumulative incidence of the first ever appropriate ICD therapy (ATP or shock)

The cumulative incidences of appropriate device therapy were 18%, 25%, and 34%, at 2, 4, and 10 years, respectively. As shown in Fig. 1, the risk for appropriate ICD therapy was highest during the first 2 years after implantation and persisted during the long-term follow-up. The median interval to the first any appropriate ICD intervention was 1.1 years (1.6–6.0 years). The first appropriate ICD intervention was shock delivery as initial therapy in 48 patients and ATP in 43 patients. The mean cycle length of shocked ventricular tachyarrhythmias was 231 ± 41 ms, and the mean cycle length of ventricular tachyarrhythmias treated by ATP was 307 ± 34 ms (*P* < 0.001).

**Fig. 2:**
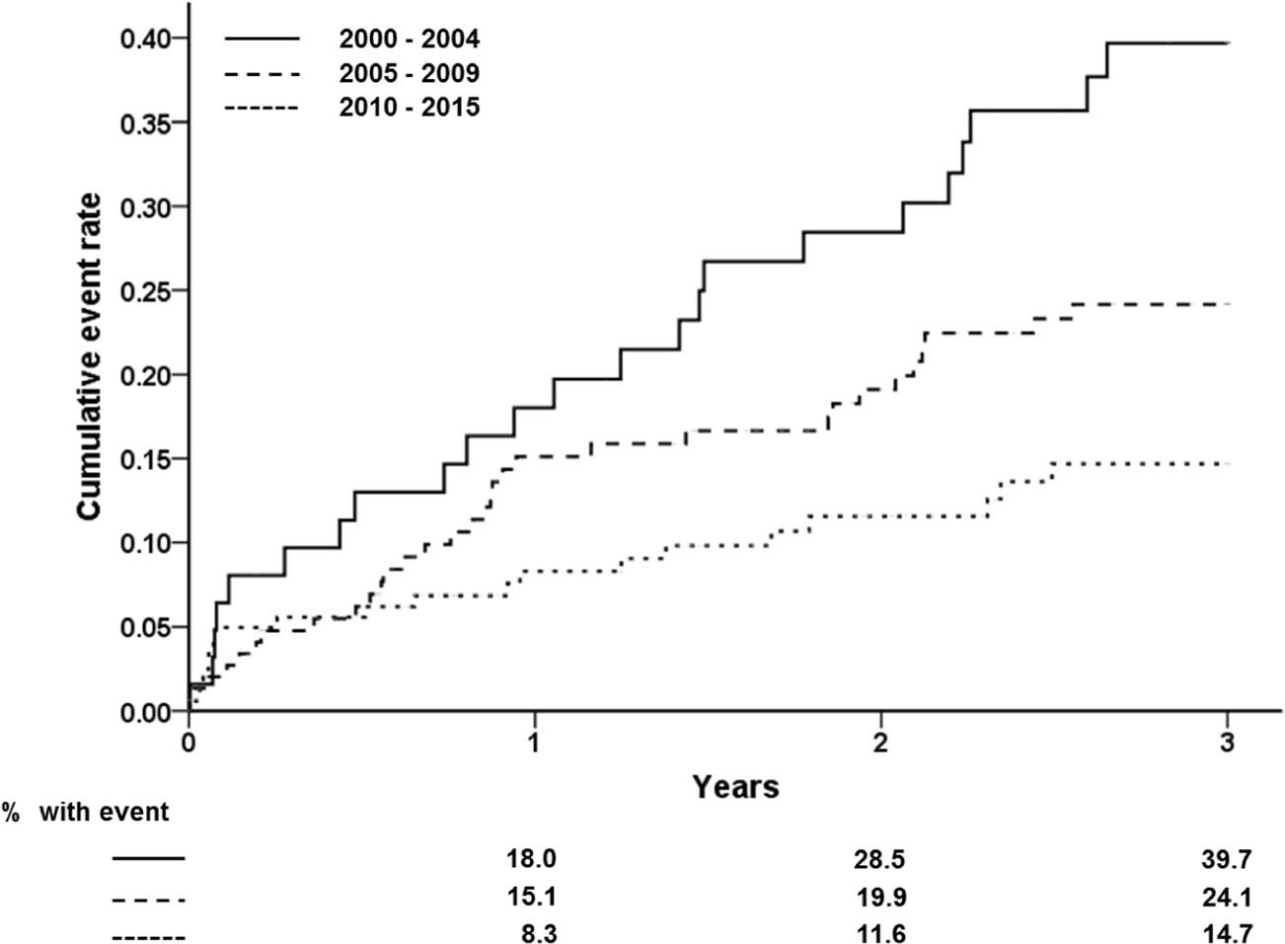
Cumulative incidence of the first ever appropriate ICD therapy (ATP or shock) stratified by 3 groups of date of ICD implantation. Solid line, implantation 2000–2004; long dashed line, implantation 2005–2009; short dashed line, implantation 2010–2015

In Fig. 2, the temporal trend of appropriate ICD therapy by the implant period is presented. At the 3-year follow-up, cumulative appropriate ICD therapy is higher in patients implanted before 2005 than those implanted later (39.7% versus 24.1% and 14.7%; both *P*’s < 0.05). The mean cycle length of ventricular tachyarrhythmias triggering the first appropriate ICD therapy was not different between the different implant periods (*P* = 0.70).

### Ventricular tachyarrhythmias triggering ICD intervention

In total, 690 episodes with appropriate ICD intervention (91 patients) were adjudicated. The number of episodes ranged from 1 to 95 per patient. The majority of patients (70%) experienced less than 5 episodes with appropriate ICD interventions. Table 2 shows characteristics of the episodes with appropriate ICD therapy. Four hundred ninety-five episodes for which ATP was delivered as the first therapy were recorded in 59 patients (range 1 to 72 episodes per patient). Subsequent shock delivery was necessary in 132 (27%) of these episodes in 26 patients (range 1 to 70 episodes per patient). Shock as initial therapy was delivered in 195 episodes (62 patients; range 1 to 19 per patient).

**Table 2:** Characteristics of ventricular tachyarrhythmias by therapy type

	ATP only (*n* = 363)	ATP + shock (*n* = 132)	Shock only (*n* = 195)
No. of patients	51	26	62
Patients with ≥ 5 VTa	18 (29%)	4 (15%)	14 (23%)
VTa cycle length, ms	341 ± 48	285 ± 40	229 ± 39
VTa cycle length ≤ 300 ms	61 (17%)	86 (65%)	195 (100%)
Morphology of VTa			
Monomorphic	363 (100%)	130 (98%)	129 (66%)
Polymorphic	–	2 (2%)	66 (34%)

*ATP*, antitachycardia pacing; *VTa*, ventricular tachyarrhythmia

**Fig. 3:**
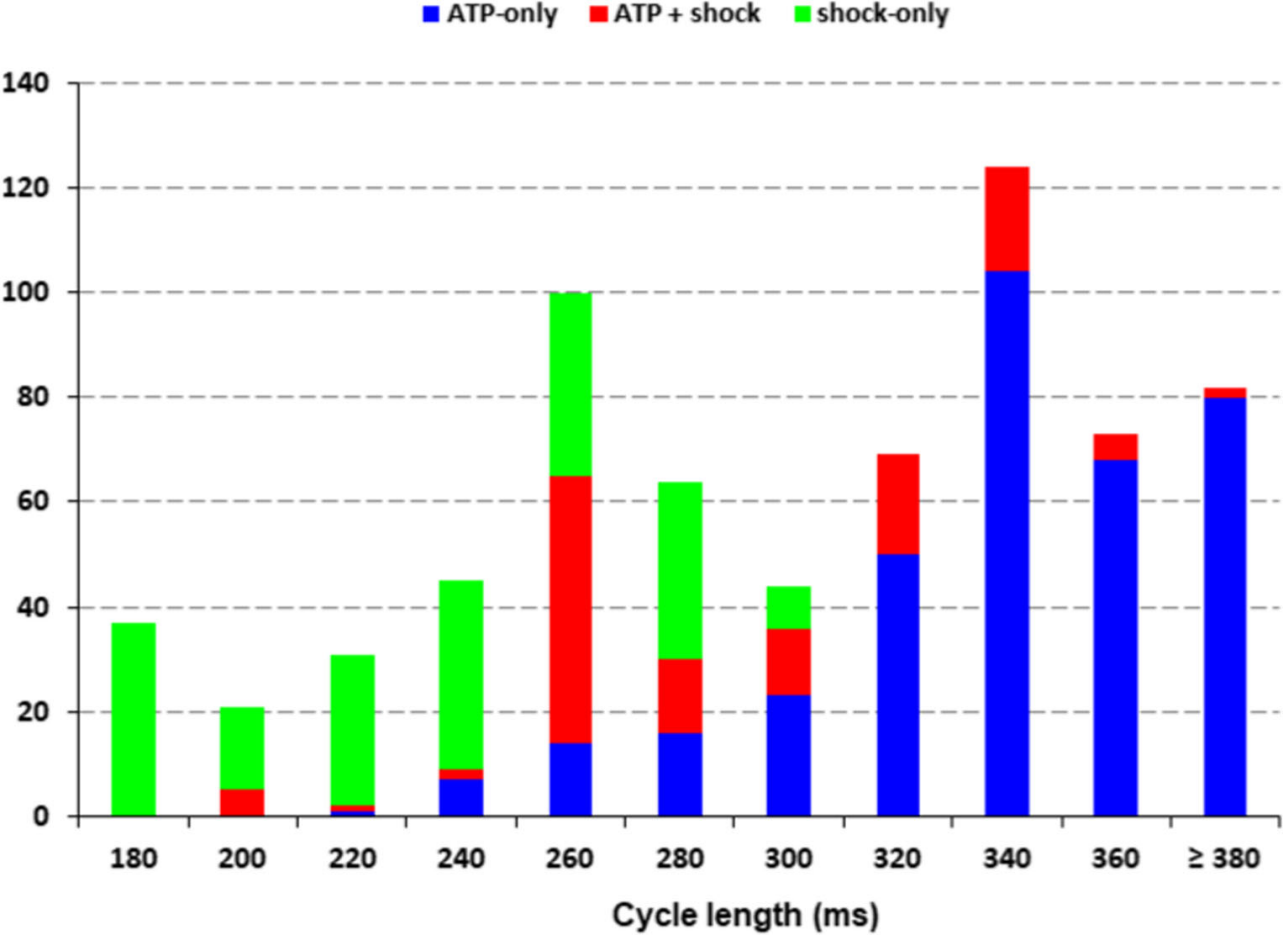
Distribution of ventricular tachyarrhythmias by initial rate

Figure 3 presents the distribution of cycle lengths of ventricular tachyarrhythmias for which ICD therapy was delivered. The mean cycle length of shocked arrhythmias was shorter compared to those treated with ATP (229 ± 39 ms versus 326 ± 52 ms; *P* < 0.001). When considering a cutoff value of 300 ms for cycle length, the majority of arrhythmias with cycle length ≤ 300 ms (82%) were treated by shock, either as initial therapy or after unsuccessful ATP. In contrast, arrhythmias with cycle length > 300 ms were mainly (83%) treated by ATP only. The association between clinical covariates and ventricular arrhythmias with cycle length > 300 ms in univariate and multivariate logistic regression analyses is shown in Table 3. The presence of a remote myocardial infarction (OR 2.08; 95% CI 1.08–3.97) and LVEF ≤ 0.35 (OR 2.09; 95% CI 1.09–4.00) were significantly associated with the occurrence of ventricular tachyarrhythmias with cycle length > 300 ms which are mainly terminated by ATP. When considering the implant period, ventricular arrhythmias with cycle length > 300 ms were less likely to occur in patients implanted after 2010 (OR 0.29; 95% CI 0.12–0.69).

**Table 3:** Clinical covariates associated with ventricular arrhythmias (cycle length > 300 ms) in univariate and multivariate analyses

Clinical covariates	Univariate			Multivariate		
	OR	95% CIs	*P* value	OR	95% CIs	*P* value
Age (years)	1.01	0.99–1.03	0.42	–	–	–
Male gender	0.99	0.48–2.05	0.98	–	–	–
NYHA class > II	2.01	0.71–5.65	0.19	–	–	–
LVEF ≤ 0.35	2.37	1.26–4.49	0.008	2.09	1.09–4.00	0.03
Remote myocardial infarction	2.36	1.25–4.45	0.008	2.08	1.08–3.97	0.03
Implant period						
2000–2004 (ref)	–	–	–	–	–	–
2005–2009	0.46	0.22–0.97	0.04	0.33	0.24–1.14	0.11
2010–2015	0.23	0.10–0.54	0.001	0.29	0.12–0.69	0.006

*CIs*, confidence intervals; *OR*, odds ratio; *LVEF*, left ventricular ejection fraction; *NYHA*, New York Heart Association

### Mortality

During follow-up, 66 patients (18%) died. At 10-year follow-up, the observed cumulative mortality was 35%. The median interval from device implantation to death was 2.7 years (1.6–6.0 years). In univariate analysis, no association between any appropriate device therapy (ATP or shock) and mortality was observed (*P* = 0.45). When considering appropriate ICD shocks, a similar result was found (*P* = 0.41).

## Discussion

In the current study, we show the rate distribution of ventricular tachyarrhythmias and type of delivered ICD therapy, in patients who survived OHCA-VF. The main findings are (1) the cumulative incidence of appropriate ICD therapy at 10 years was 34%, (2) the majority of patients (70%) experienced less than 5 ventricular tachyarrhythmias treated with ICD therapy, (3) ventricular tachyarrhythmias with cycle length ≤ 300 ms are mainly terminated by shock, and (4) the presence of LVEF ≤ 0.35 and a remote myocardial infarction are associated with slower ventricular tachyarrhythmias (cycle length > 300 ms).

Although prophylaxis of ICD therapy has been widely studied, there is relatively little data on the outcome of patients with a secondary prevention indication. The incidence of appropriate ICD therapy in a secondary prevention population has been studied in a few studies. Freedberg et al. [15] observed appropriate ICD therapy in 62% of patients after the 2-year follow-up. In the Leiden registry, cumulative incidences of appropriate ICD therapy were 52% and 61%, at 5 and 10 years, respectively [16]. More recently, Schaer et al. [17] reported a cumulative incidence of appropriate ICD therapy of 65% at 10 years. The incidence of appropriate ICD therapy in our study (33.5% at 10 years) is lower compared to the previous studies. The higher incidences in previous studies can be explained by the heterogeneous group of patients with both VF and VT as index arrhythmia. A post hoc analysis of the Antiarrhythmics Versus Implantable Defibrillators (AVID) trial compared the incidence of ICD therapy according to the index arrhythmia [18]. After the 3-year follow-up, patients with VT as index arrhythmia were more likely to experience appropriate ICD therapy than those with VF as index arrhythmia (75.5% versus 47.4%). In a recent cohort analysis of 239 patients with a median follow-up of 7.8 years, Boule et al. [19], patients presenting with VF were less likely to require appropriate ICD therapy compared to those presenting with VT (sub-hazard ratio 0.62). Patients who were implanted after an aborted cardiac arrest due to VF have a lower risk of appropriate ICD therapy during the follow-up compared to those with VT as index arrhythmia.

Studies reporting on the type of ventricular arrhythmias during follow-up in secondary prevention patients are scarce. Post hoc analysis of the Pacing Fast VT Reduces Shock ThErapies (PainFREE Rx II) trial and the Inhibition of Unnecessary RV Pacing with AV Search Hysteresis in ICDs (INTRINSIC RV) trial demonstrated that the rate distribution of ventricular arrhythmias was similar between the primary and secondary prevention patients [20, 21]. However, the majority of secondary prevention patients had VT as index arrhythmia in both trials. In our study, only patients who survived OHCA-VF were included to assess the eligibility for the S-ICD. Patients with known recurrent monomorphic VT thought to be amenable for ATP termination are considered not eligible for the S-ICD. The presence of monomorphic VTs is more common late after myocardial infarction due to reentry around scar [22]. In the present study, slower ventricular arrhythmias (rate < 200 bpm) were associated with the presence of LVEF ≤ 35% and a remote myocardial infarction.

The armamentarium of devices that defibrillate in order to prevent SCD has expanded in recent years. As a consequence, ICDs have been used in patients with a variety of clinical needs, including those with documented monomorphic VTs, survivors of OHCA-VF, patients with requirements for bradycardia pacing or cardiac resynchronization with a concomitant indication for an ICD, and patients at increased risk for SCD. Given the short- and long-term complications of ICDs, it is important to select the right device carefully for each individual patient. In clinical practice, many physicians think that a device without ATP to be inferior. However, the perceived requirement of ATP must be balanced with the benefit of S-ICD therapy whose risk of any VT is low for the first years after implantation. Considering recurrent ventricular arrhythmias in S-ICD patients, a recent analysis of the EFFORTLESS registry demonstrated that only 2.2% of patients experienced ICD therapy for more than 1 episode of monomorphic VT [23]. In addition, only 0.5% of patients had the S-ICD removed for conceived need for ATP. Taken all together, the implantation of a S-ICD might be reasonable in survivors of OHCA-VF with LVEF > 35% without the presence of a remote myocardial infarction.

## Limitations

Our study has several limitations. First, the study is a retrospective, single-center study. However, data in this ICD registry is prospectively collected. Another possible limitation is that the risk of appropriate ICD therapy depends on ICD programming. Over the years, programming of higher rate cutoffs for detection and longer detection duration to reduce unnecessary ICD interventions has been recognized both in the primary and secondary preventions [24, 25]. We accounted for this by defining 3 groups according to date of implant. According to this, the reported overall incidence of appropriate ICD therapy in the present study is probably overestimated rather than underestimated. Last, the negative impact of shocks on mortality has been consistently seen in trials with the primary prevention patients. We found no association between shocks and mortality. However, our study was not designed to evaluate the association between shocks and mortality. The impact of shocks on mortality in patients with a secondary prevention indication merits further investigation.

## Conclusion

The findings of this study suggest that the implantation of the S-ICD may be reasonable in survivors of OHCA-VF with LVEF > 35% without a remote myocardial infarction.
